# Skeletal muscle characteristics are preserved in hTERT/cdk4 human myogenic cell lines

**DOI:** 10.1186/s13395-016-0115-5

**Published:** 2016-12-08

**Authors:** Matthew Thorley, Stéphanie Duguez, Emilia Maria Cristina Mazza, Sara Valsoni, Anne Bigot, Kamel Mamchaoui, Brennan Harmon, Thomas Voit, Vincent Mouly, William Duddy

**Affiliations:** 1 INSERM, CNRS, Institute of Myology, Center of Research in Myology, Sorbonne Universities, UPMC Univ Paris 6, Paris, France; 2 Northern Ireland Centre for Stratified Medicine, Altnagelvin Hospital Campus, Ulster University, Londonderry, Northern Ireland UK; 3 Department of Life Sciences, Center for Genome Research, University of Modena and Reggio Emilia, Modena, Italy; 4 Research Center for Genetic Medicine, Children’s National Medical Center, Washington, DC 20010 USA; 5 NIHR Biomedical Research Centre, Institute of Child Health, University College London, 30 Guilford Street, London, UK

## Abstract

**Background:**

hTERT/cdk4 immortalized myogenic human cell lines represent an important tool for skeletal muscle research, being used as therapeutically pertinent models of various neuromuscular disorders and in numerous fundamental studies of muscle cell function. However, the cell cycle is linked to other cellular processes such as integrin regulation, the PI3K/Akt pathway, and microtubule stability, raising the question as to whether genetic modification related to the cell cycle results in secondary effects that could undermine the validity of these cell models.

**Results:**

Here we subjected five healthy and disease muscle cell isolates to transcriptomic analysis, comparing immortalized lines with their parent primary populations in both differentiated and undifferentiated states, and testing their myogenic character by comparison with non-myogenic (CD56-negative) cells. Principal component analysis of global gene expression showed tight clustering of immortalized myoblasts to their parent primary populations, with clean separation from the non-myogenic reference. Comparison was made to publicly available transcriptomic data from studies of muscle human pathology, cell, and animal models, including to derive a consensus set of genes previously shown to have altered regulation during myoblast differentiation. Hierarchical clustering of samples based on gene expression of this consensus set showed that immortalized lines retained the myogenic expression patterns of their parent primary populations. Of 2784 canonical pathways and gene ontology terms tested by gene set enrichment analysis, none were significantly enriched in immortalized compared to primary cell populations. We observed, at the whole transcriptome level, a strong signature of cell cycle shutdown associated with senescence in one primary myoblast population, whereas its immortalized clone was protected.

**Conclusions:**

Immortalization had no observed effect on the myogenic cascade or on any other cellular processes, and it was protective against the systems level effects of senescence that are observed at higher division counts of primary cells.

**Electronic supplementary material:**

The online version of this article (doi:10.1186/s13395-016-0115-5) contains supplementary material, which is available to authorized users.

## Background

Research on neuromuscular disorders, including potential therapeutic options, depends on the careful observation of clinical symptoms and of biopsy material from human subjects, and also on the availability of disease models that both accurately reflect aspects of the pathology and facilitate experimental intervention. Animal models allow the experimental manipulation of fully vascularized, innervated muscle tissue, and they often recapitulate to a large extent the complexity of interactions between human cell and tissue types, and how those interactions change in disease and development. In contrast, the relative homogeneity of isolated and purified cell lines has a double-edged significance: it renders them pertinent only to certain aspects of certain pathologies, but it also facilitates the close study of specific molecular mechanistic events. In addition, where they are understood to closely recapitulate some measurable aspect of the pathology, cell models can be highly amenable to high-throughput studies.

From a systems biology perspective, compared with whole organisms, cell lines more closely (however imperfectly) represent a single enclosed apparatus in which changes to one or more component(s) have direct mechanistic impact on connected components. This is particularly true of pathologic muscle, in which processes such as regeneration, inflammation, fibrosis, and adipogenesis all conspire to a general loss of order and increase in tissue heterogeneity. These changes in whole muscle composition can be observed in transcriptomes and other omics profiles, and may obscure underlying mechanistic details. However, isolated primary myoblasts suffer the disadvantage that they undergo senescence with amplification in tissue culture. Immortalization avoids senescence and thereby facilitates subsequent cloning to select a highly pure model cell line.

Adult human primary myoblasts senesce after approximately 25 rounds of division in tissue culture due to cell cycle suppression by the p16^Ink4a^-dependent stress pathway and progressive telomere shortening which triggers cell cycle exit mediated by activation of p53 [1–3]. We showed that immortalization of human myoblasts requires bypassing of both of these senescence mechanisms, and we achieved this by transduction of the murine cyclin-dependent kinase (cdk)-4, which overcomes the p16 pathway, and of human telomerase reverse transcriptase (hTERT) which preserves telomere length [4].

Using this method, we have created a large collection of immortalized human myoblasts isolated from a wide range of neuromuscular disorders. Several have been validated as experimental models for Duchenne muscular dystrophy (DMD) [5–8], limb girdle muscular dystrophy type 2B (LGMD-2B) [9], facioscapulohumeral muscular dystrophy (FSHD)—including mosaic-origin control lines from the same patient [10–12], and excitation-contraction coupling and calcium homeostasis [13]. These cell lines have contributed to the development of therapeutic approaches such as oligonucleotide-mediated exon skipping [5], read-through of non-sense mutations [6], and gene correction [7, 8] for DMD, to the study of ryanodine receptor 1 (RyR1) deficiency in congenital myopathies [14], cell senescence in myotonic dystrophy type I [15], the involvement of IL-6 and Akt in the pathogenesis of myasthenia gravis [16], the dysregulation of DUX4c [11] and the role of FAT1 [12] in FSHD, and the shutdown of quiescence pathways in ageing [17]. They have also been used to explore fundamental aspects of muscle cell physiology including: the role of β-arrestins in myogenesis [18], the role of MMP-14 in human myoblast collagen invasion [19], nuclear protein spreading between nearby myonuclei [20], the effects of oxidative stress on myoblast calcium-dependent proteolysis [21] and the proteome [22], engineering of 3D micro-muscles [23], and the function of miRNAs during myoblast differentiation [24], this list being non-exhaustive. Thus, they have become an important resource to the muscle research community.

To validate the use of immortalized myoblasts, we previously confirmed the expression of myogenic markers MyoD, NCAM, desmin, and various myosin isoforms, in differentiated myotubes, the morphology of myotubes by immunostaining of sarcomeric myosin, and their capacity to contribute to myofibre formation in vivo [4, 25]. A separate validation was carried out on two lines of healthy and four of dysferlin-deficient (LGMD-2B) immortalized clones: relative to their parent primary populations, they showed unaltered expression of myogenic markers MHC, alpha-tubulin, desmin, and caveolin-3, unaltered sarcomere formation and subcellular localization on immunofluorescence imaging of these same markers, and unaltered membrane repair processes [9]. In a third study, the physical properties of Ca2^+^ release and its response to pharmacological intervention were unaltered in immortalized lines compared with primaries [13].

However, the cell cycle is a major pathway that is linked to other cellular processes, including integrins [26], the PI3K/Akt pathway [27], apoptosis [28], and microtubule stability [29], each of which may be important to muscle cell function and pathology. Perturbations to the cell cycle may impact on these and on other less directly associated processes. Furthermore, it is increasingly understood that cyclin-dependent kinases are implicated in roles beyond the cell cycle, including aspects of transcription, metabolism, and stem cell self-renewal (reviewed [30]). Despite the wide use of our cdk4/hTERT immortalized human myoblasts, the potential secondary effects of immortalization have been tested only for those parameters listed above, relating to myogenesis and the functioning of membrane repair and calcium homeostasis. Secondary effects of cdk4/hTERT transduction on other processes that have not yet been tested would challenge the usefulness of these immortalized lines as experimental models and could undermine previous and ongoing studies that use these cell lines.

A second risk to the representativeness of these cell models occurs when we select a clonal line from the immortalized population: despite that the purity of our primary cell isolates is monitored by immunostaining against the myogenic marker desmin, the immortalized population may still exhibit cell-to-cell variability, so that any given clone may represent only part of its parent primary population. A third risk is that, relative to primary populations, immortalized clonal lines that undergo long-term experimental use are repetitively amplified and maintained for prolonged periods in tissue culture conditions, whereas time in tissue culture has been associated to loss of myogenic potential [31–33]. Thus, it is important to determine whether immortalized clonal myogenic lines diverge from their mortal parent primary populations.

Here we use extensive transcriptomic profiling of cdk4/hTERT immortalized human myoblasts in their undifferentiated and differentiated states, comparing them with their mortal parent populations, and with a non-myogenic reference population. In this way, we obtain a systems level view of cellular processes, allowing us to determine whether secondary effects are incurred by cdk4/hTERT immortalization.

## Results

### Immortalized clonal lines retain the system profile of their primary parent population

**Fig. 1:**
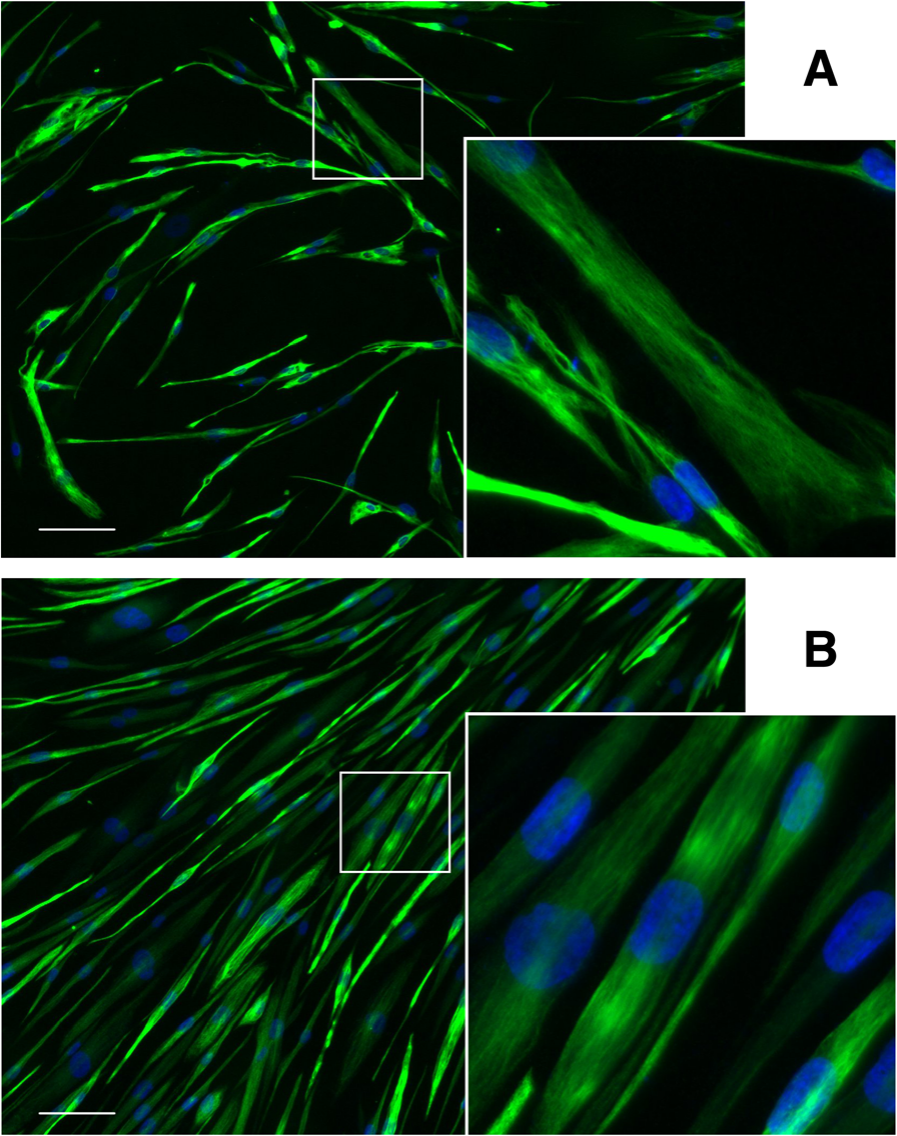
Representative images of primary and immortalized human myotubes. Myogenic cells were purified by magnetic bead sorting of CD56 expression then differentiated for 5 days. Examples here are from primary (**a**) and immortalized (**b**) populations of a healthy subject (CHQ). Myotubes were immunostained for desmin (*green*) to determine the percentage of myogenic purity (>94% for all samples). Magnified region shows the structure of desmin filaments in both primary and immortalized cells. Nuclei are DAPI-stained (*blue*). Scale = 100 μm

We generated microarray gene expression profiles for 94 samples comprising primary myoblasts and their corresponding immortalized clones in both differentiated and undifferentiated states (average of 4 cell culture replicates each) from 5 human subjects (Table 1; 2 healthy and 3 with Duchenne muscular dystrophy—DMD), together with primary populations of non-myogenic (CD56-ve) cells from the muscles of 8 other human subjects. Prior to analysis, each of the populations was sorted for CD56 using magnetic beads and confirmed to be >94% desmin-positive for myogenic cells (Fig. 1) or 100% desmin-negative for the CD56-ve populations. To maximize the maturity and the expression levels of late myogenic markers, myotubes were maintained for 9 days in differentiation conditions, at which time no notable cell death or detachment had occurred.

**Table 1:** Cell populations used, with their origins and division counts

Cell line	Phenotype	Mutation	Age of subject	Sex of subject	Muscle of origin	Division counts^a^ (prim./immort.)
CHQ	Healthy	–	5.5 days	Female	Quadriceps	29/83
C25	Healthy	–	25 years	Male	Semitendinosus	16/86
DMD6594	Duchenne MD	Del 48–50	20 months	Male	Quadriceps	7.5/51
DMD6311	Duchenne MD	Del 45–52	23 months	Male	Quadriceps	10/50
DMD8036	Duchenne MD	Del 48–50	6 years	Male	Biceps	8.5/46

^a^Number of divisions undergone by primary and immortalized clonal cells at the time of harvesting for transcriptomic analysis

**Fig. 2:**
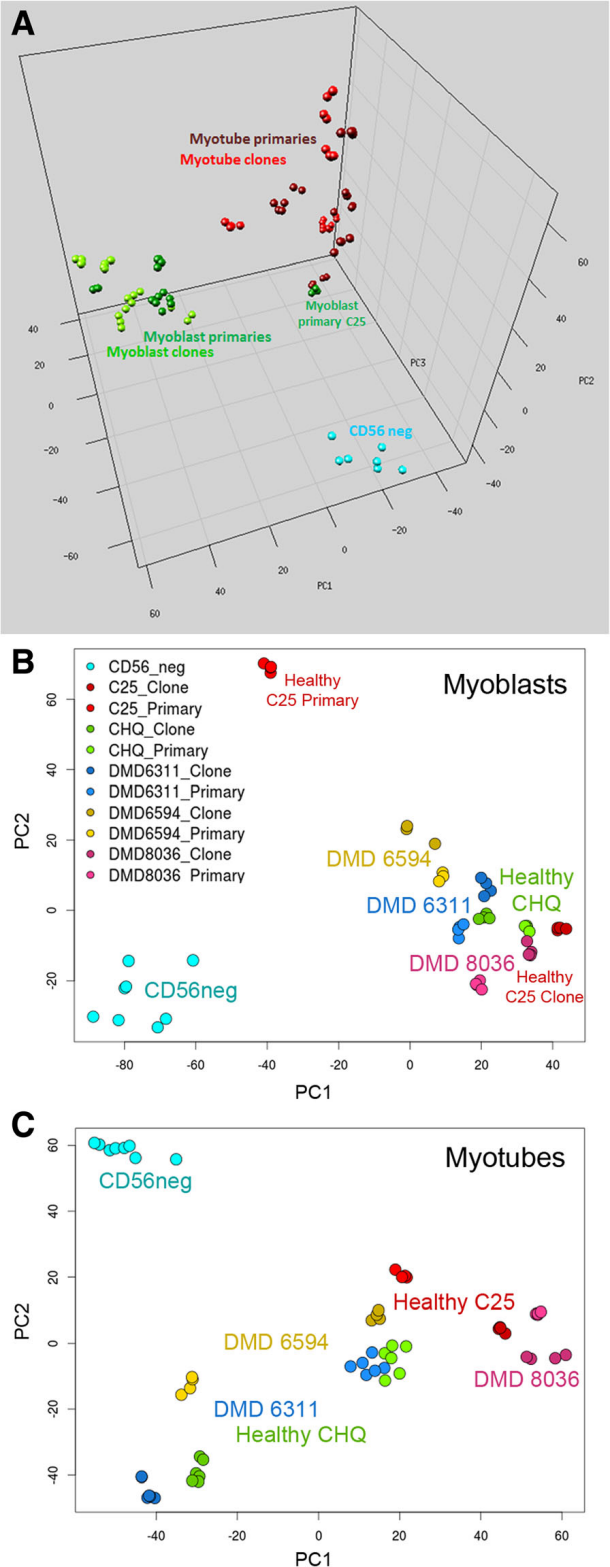
Principal component analysis of gene expression data from primary cells and immortalized clones of human myoblasts and differentiated myotubes, and from primary non-myogenic (CD56-negative) muscle-resident cells. **a** Immortalized myoblasts (*light green*) cluster together with primary myoblasts (*dark green*) and immortalized myotubes (*light red*) together with primary myotubes (*dark red*). Both are separated from non-myogenic cells (*cyan*). Data-points are projected onto principal components 1, 2, and 3. **b** and **c** The same data for myoblasts (**b**) and myotubes (**c**) alone, projected onto principal components 1 and 2. For myoblasts, PC1 accounted for 43% and PC2 for 17% of the total variance in the data. For myotubes, PC1 accounted for 32% and PC2 for 22% of total variance. Each population is labelled and colored separately according to the key presented in (**b**)—immortalized clone populations are colored a darker shade than their respective primaries. Each immortalized clone clustered near to its parent primary population, and clones were not shifted in any particular direction relative to their parent primary population. Each data-point corresponds to a separate culture dish (average *n* = 4 dishes per cell line)

Data complexity reduction using principal component analysis (PCA) showed that samples clustered into three groups: myoblasts and myotubes were separated from each other across principal components 1 and 2, whereas the CD56-negative population was separated from the others by principal component 3 (Fig. 2). An overall view of the three groups, with primaries and clones indicated, is presented (Fig. 2a), and the same data are shown again for myoblasts (Fig. 2b) and myotubes (Fig. 2c) alone (for comparison, CD56-neg are included in each case). Replicate culture dishes clustered tightly. Importantly, immortalized clones clustered closely to their parent primary populations, and data-points representing clones were not shifted in any particular direction relative to their parent primary population. Among the myoblasts, the primary C25 population was an outlier, clustering separately from the other myoblast populations (including from its own immortalized clonal line)—this primary population is analyzed and discussed further below.

### Differentiation of immortalized clonal lines follows the normal myogenic cascade

**Fig. 3:**
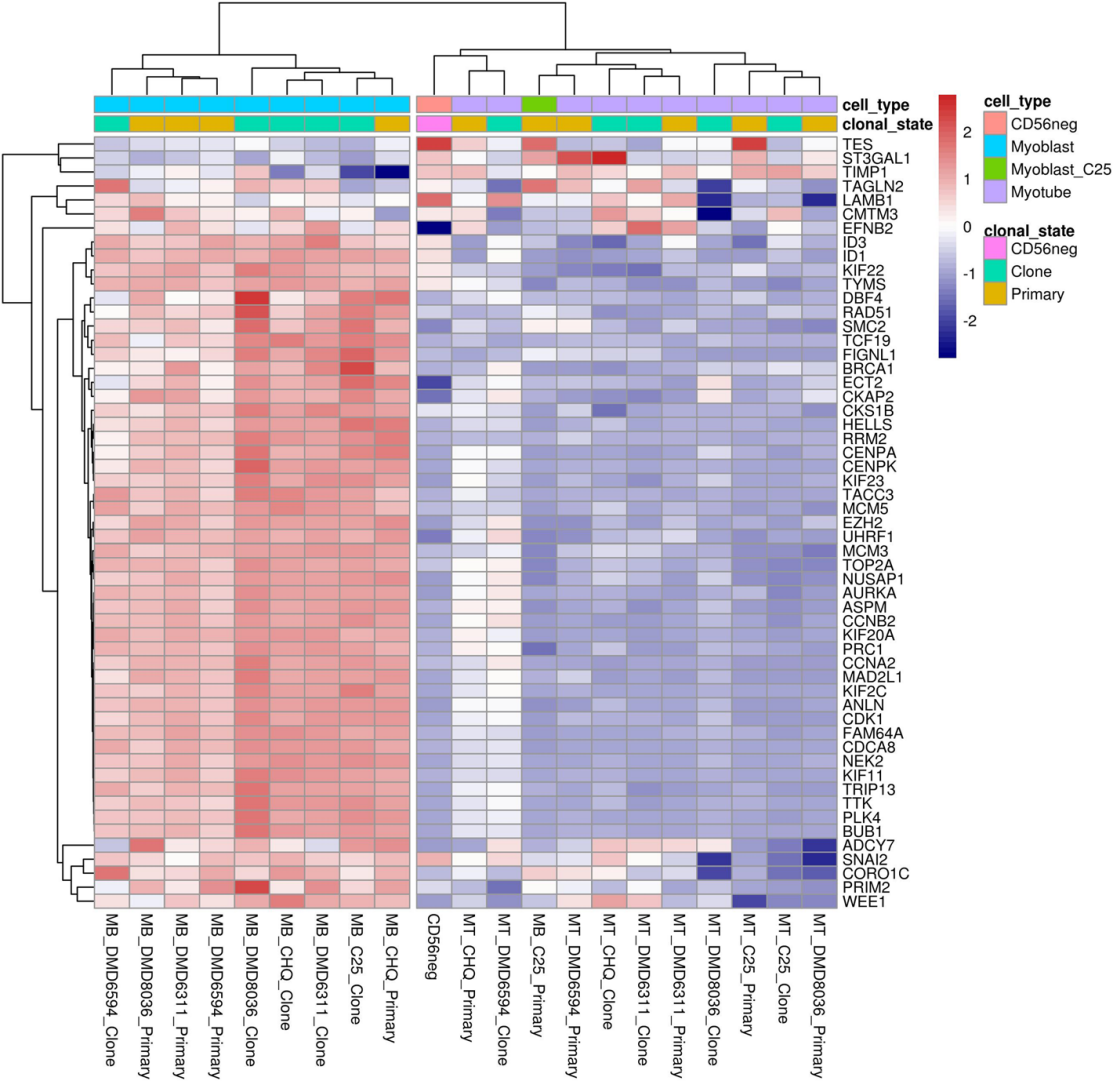
Heatmap showing the expression levels in immortalized and primary myogenic human lines of genes that are consistently and strongly downregulated in a panel of previous studies of myoblast differentiation. A gene is shown if it was among the 300 most strongly downregulated after five or more days of differentiation in at least three of four studies of human and mouse primary myoblasts, and C2C12 myoblasts. Expression values are *row scaled* (i.e., changes are relative across each row for ease of interpretation). Hierarchical clustering analysis of the cell lines, indicated by branches at top, places myotube lines (*indicated in lilac in the cell_type bar at top*) separately from myoblasts (*cyan cell_type*), except for the C25 primary cells (*green cell_type*). The non-myogenic population (*orange cell_type*) clusters with the myotubes. Immortalized clones (*green clonal_state*) show the same switch from myoblast to myotube as their parent primary populations (*yellow clonal_state*). Hierarchical clustering analysis was also applied to genes (*branches to left*)

To study the behavior of genes involved in the myogenic cascade in immortalized and primary cell lines, we created a consensus set of genes that were differentially expressed in four previously published studies of myoblast differentiation (Additional file 1: Table S1). These studies included human and mouse primary myoblasts, and C2C12 myoblasts, and we retained genes that were strongly up or downregulated after five or more days of differentiation in at least three of the four datasets. For the consensus downregulated genes, a heatmap showing their relative expression levels across our samples is shown: note the rows near the top of the figure that indicate the *cell_type* and *clonal_state* (Fig. 3). Hierarchical clustering analysis of the samples is presented on the same plot (branch lines at top of figure) and shows that these genes neatly separated myoblasts (cyan in the cell_type row) from myotubes (lilac in the cell_type row) with no effect of immortalization (green and yellow in the clonal_state row)—myoblast clones showed similar expression values to the myoblast primary populations, and myotube clones showed similar expression values to the myotube primary populations.

Differentiation induced consistent downregulation in both primaries and immortalized clones of genes involved in cell cycle regulation and DNA replication, such as centromere components (CENPA, CENPK), mini-chromosome maintenance (MCM3), DNA topoisomerases (TOP2A), regulation of mitotic spindle formation (CDCA8), G2/mitotic-specific cyclin-B2 (CCNB2), and the MyoD regulator ID2 [34]. Genes such as cyclin-dependent kinase 1 (CDK1) and cyclin dependent kinase regulators (e.g., CKS1B) were downregulated normally in clonal lines despite the presence of the murine cdk4 transgene.

**Fig. 4:**
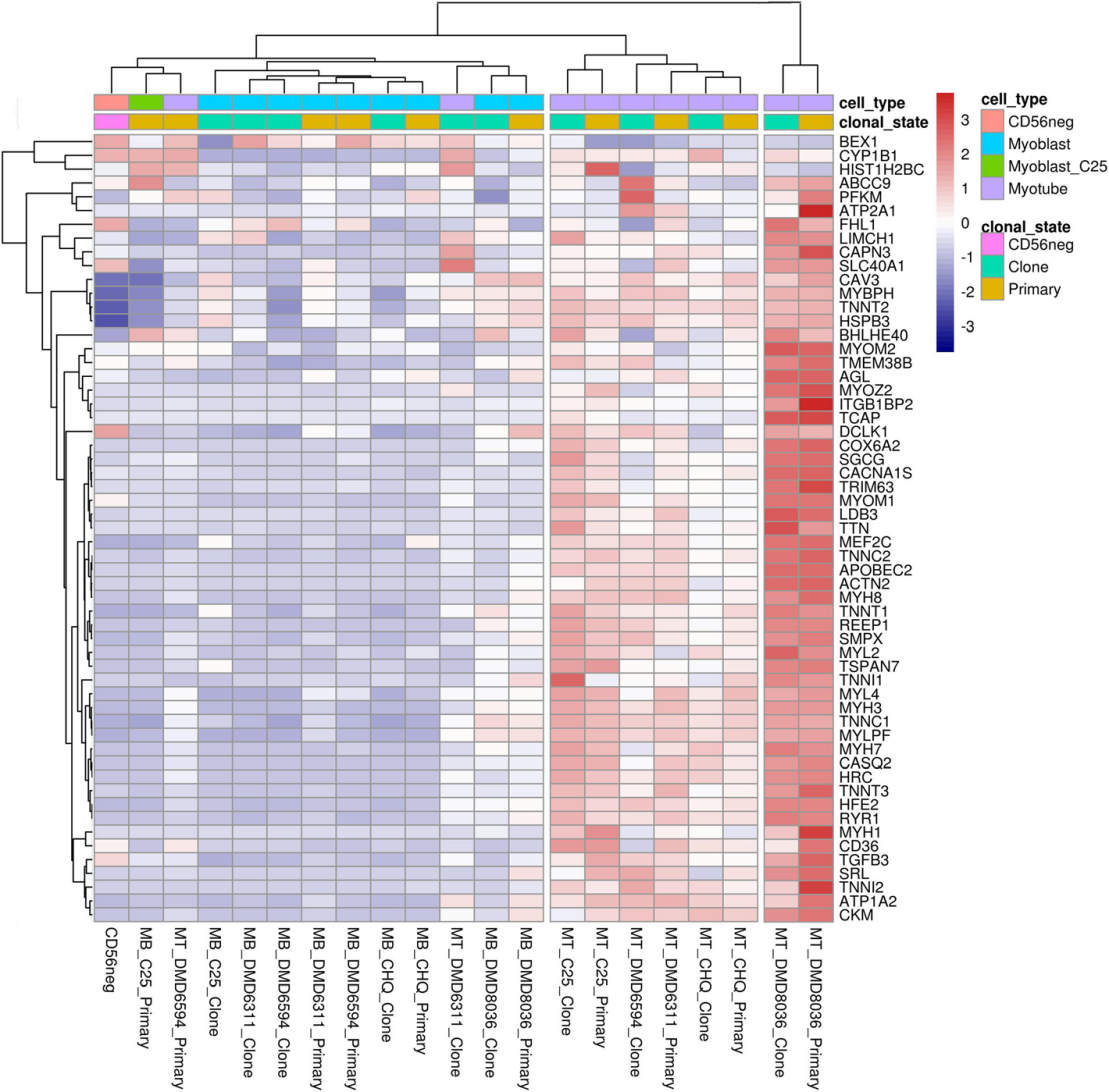
Heatmap showing the expression levels in immortalized and primary myogenic human lines of genes that are consistently and strongly upregulated in a panel of previous studies of myoblast differentiation. A gene is shown if it was among the 300 most strongly upregulated after five or more days of differentiation in at least three of four studies of human and mouse primary myoblasts, and C2C12 myoblasts. Expression values are *row scaled*. Hierarchical clustering analysis of the cell lines and genes are indicated by branches at top and left, respectively. Cell type and differentiation state are indicated by the *cell_type bar* at top. Immortalized clones, primary myogenic cells, and non-myogenic cells are indicated by the *clonal_state bar* at top

For the consensus upregulated genes, a similar heatmap is shown (Fig. 4). Similarly to the consensus downregulated genes, immortalization had no effect on the upregulation of the myogenic regulator MEF2C, metabolic genes such as creatine kinase (CKM) and ATPase 1A2 (ATP1A2), nor on contractile components and their regulators, including alpha-actinin (ACTN2), calpain-3 (CAPN3), myosins and their partners (MYBPH, MYL2, MYL4, MYLPF, MYH1, MYH3, MYH7, MYH8), troponins (TNNC1, TNNC2, TNNI1, TNNI2, TNNT1, TNNT3), myomesins (MYOM1, MYOM2), myozenin 2 (MYOZ2), and titin (TTN). Markers of mature myotube formation such as the dihydropyridine receptor calcium channel (CACNA1S), and the RYR1, were also unaffected by immortalization.

There were few exceptions to the clustering of myoblasts separately from myotubes. The myotubes (both primary and immortalized) of one subject (DMD8036) showed stronger upregulation of myogenic genes and clustered separately from the other myotubes. The outlier myoblast primary line (C25) clustered with myotubes in the heatmap for downregulation, suggesting that this line had shutdown its cell cycle. The myotubes of two lines (DMD6311 clones and DMD 6594 primaries), despite strong downregulation of the cell cycle, showed relatively weak upregulation of the myogenic cascade and clustered with myoblasts in the upregulated heatmap. As a general rule, the normal myogenic cascade was maintained in immortalized clonal lines.

### Immortalization has no effect on other systems processes and has no effects that are similar to previously reported perturbations of muscle cells

We used rank-based gene set enrichment (GSEA) of 2784 canonical pathways and gene ontology terms to test for effects of immortalization on systems processes, separately in myoblasts and myotubes. Immortalized clones of neither myoblasts nor myotubes showed any significantly enriched rank distributions with FDR *q* values < 0.05 for any of the canonical pathways and gene ontology terms. Since we expected the cdk4 transgene to elicit changes to cell cycle regulation in myoblasts, we specifically checked rank distribution patterns for related gene sets. Of 22 cell cycle-related gene sets, none were significant (all had FDR *q* values > 0.2), but 6 had nominal *p* values < 0.05 (a measure that does not adjust for gene set size or multiple hypothesis testing). As these can be considered borderline significant, we include a heatmap (Additional file 1: Figure S1) showing the expression levels of genes that are driving this effect (defined by the overlap of GSEA leading edges). These genes generally showed some upregulation in immortalized myoblast clones relative to primary myoblasts, but with the C25 primary outlier showing relatively very strong dysregulation (most genes were downregulated in C25 relative to all of the other lines and a few were upregulated).

To test for effects on gene expression that were similar to previously reported perturbations of muscle cells, we applied GSEA to muscle gene sets (http://sys-myo.rhcloud.com/muscle_gene_sets.php). These gene sets were comprised of 393 lists of genes that were up- or downregulated in a large variety of genetic and experimental comparisons in published muscle microarray studies, mainly of human or murine tissue and cell samples. These lists were obtained from more than 100 studies, and included the 4 in vitro differentiation datasets from which our consensus set was derived as described above. When immortalized clones of myoblasts or myotubes were compared to their respective primary cells, neither showed any significantly enriched rank distributions with FDR *q* val < 0.05 for any of the 393 muscle gene sets. Muscle gene sets represent an ensemble of genes that are identified empirically to respond at the expression level to muscle perturbations, so their lack of change in immortalized v primary cells is indicative that immortalization has little effect on these lines. For instance, it is informative to contrast those results with the following: when genes of our dataset were ranked according to their change in myotubes v myoblasts, GSEA on the muscle gene sets specifically identified strong overlap with 35 of the 393 muscle gene sets with a very highly stringent statistical cut-off of FDR < 0.001: these highly significant muscle gene sets are visualized using an enrichment map—this shows gene sets that were downregulated (in myotubes v myoblasts) in blue with color intensity proportional to the enrichment score (none of these highly significant gene sets were upregulated in this case), and grey connections represent the sharing of genes between gene sets (i.e., some gene sets are similar to each other) (Additional file 1: Figure S2). This analysis shows the strong similarity of previously published muscle differentiation gene expression changes with differentiation-induced changes in the present study when myotubes are compared to myoblasts—whereas the comparison of immortalized with primary cells, in contrast, did not induce changes that were similar to any previously published data.

Rank-based gene set enrichment testing of canonical pathways, gene ontology, and previous muscle studies, were all consistent with hTERT/cdk4 immortalization having a minimal effect on the systems characteristics of human myoblasts.

In addition, we tested for specific effects of immortalization on other pathways previously observed to be influenced by the cell cycle. We found no change in the levels of specific phosphorylated proteins involved in the regulation of Akt pathway activity (Additional file 1: Figure S3) or in the expression of genes involved in microtubule stability (Additional file 1: Figure S4), and heatmaps of relative gene expression levels for gene ontology terms relating to these processes show no clustering of primary samples separately from immortalized lines (Additional file 1: Figures S5, S6 and S7).

### Downregulation of the cell cycle is the main effect that distinguishes the C25 primary myoblasts as outliers

Unlike their immortalized clones, primary myoblasts of the C25 lineage clustered more closely to myotubes than to other myoblasts (Fig. 2). These cells showed shutdown of cell cycle genes relative to myoblasts (Fig. 3), but none of the upregulation of contractile components observed in myotubes (Fig. 4). Differential expression analysis and over-representation tests showed that these processes represented the strongest differences of C25 primary myoblasts with other myoblasts and with myotubes (Additional file 1: Figure S8). Our interpretation of these data relate to our previous observations that human primary myoblasts senesce in tissue culture [17, 25, 35]. At the time of harvesting for analysis, which was at 16 cell divisions for the C25 primary line, our transcriptomic data strongly indicate cell cycle shutdown for this line. This coincides with proliferative arrest, since our previously published analyses [25] indicate that the rate of mean population doubling for the C25 primary population begins to slow after 10–15 divisions, indicative of slowing of the cell cycle as the population begins to senesce. This did not occur in any of the immortalized lines, thus immortalization is protective from senescent shutdown of the cell cycle.

## Discussion

Immortalization does not confer any general dysregulation of normal myogenic processes, nor of any canonical pathways or gene ontology terms. While senescence shuts down the cell cycle of primary human myogenic isolates after a number of divisions in tissue culture, hTERT/cdk4 immortalized lines are protected, retaining the characteristics of non-senescent primary lines. Indeed, the single outlier group in our dataset was a population of primary myoblasts—whereas immortalized clones derived from that primary population remained similar to the other myoblast lines.

In the search for therapeutic strategies, it is important to have models that are representative of the in vivo nature of a pathology but that also allow close dissection of the mechanistic details of the pathology. When care is taken to understand the differences between humans and animal models [36], these models can be used to gain insight into the full complexity of the human condition such as in the cross-talk between cell and tissue types, and the relationship of disease progression to developmental changes and age. Even as advances are made in the modeling of artificial muscle tissue [23], animal models remain our only possibility to observe the effects of experimental perturbations on normally vascularized and innervated muscle tissue. However, whole muscle tissue samples are not homogenous, instead consisting of a mix of constituents including myofibers, satellite cells, extracellular matrix, vascular and nerve tissue, interstitial deposits of fibrous and fatty material, fibroblasts, fibro/adipogenic (FAP) cells, and immune cells. When transcriptomic profiles are derived from whole muscle of patients and animal models, it is impossible to separate minor gene dysregulation in myonuclei (representing the bulk of the tissue) from large expression changes in a lesser component of the tissue. For instance, if immune genes are upregulated this may reflect a response of the myofibres themselves and/or an increased infiltration of immune cells. At worst, as much can be told from the whole muscle transcriptome as can be guessed from simple microscopy of a stained muscle section. In these cases, the transcriptome becomes just another measure of disease progression, because any insight into the underlying molecular mechanisms is hidden by changes in tissue composition. Laser capture microscopy can be applied to obtain pure tissue from muscle sections [37, 38], but it is highly laborious to obtain sufficient material by this method and it is rarely used.

Cell models cannot represent the more complex whole organism interactions described above, which is why a full understanding of a pathology will likely require multiple types of model. However, from the systems biology perspective, isolated cells can represent a relatively homogenous sample type so that changes in the transcriptome or in other omics profiles can be assumed to reflect changes in the mechanistically linked components of a single cellular system. So long as it is understood that an omics profile reflects an averaging across the population of harvested cells (except if single cell methods are applied), this assumption can be applied to carefully purified cell subpopulations such as the desmin-positive primary myoblast lines used in our study. To remain as close as possible to the human pathology (e.g., for the purpose of assessing therapeutic strategies), ideally human cell isolates are used. However, adult human myoblasts quickly senesce during in vitro culture [2–4, 35]. Our recent work underlines the grave importance of this in the interpretation of experimental data [17]. Immortalized lines require genetic interference with the cell cycle, but we developed these lines in order to have an inexhaustible source of cell model material, and we have continued in their use because we did not observe morphological or other obvious abnormalities, and because of pressing needs in therapeutic development. However, question-marks had remained as to whether immortalization has knock-on effects on other cellular processes that would overwhelm their natural biological variation and thus challenge their usefulness as experimental models. The results presented here show that immortalized cells retain gene expression patterns that are characteristic of their cell type, as distinct from the non-selected (CD56-ve) fraction, and whether as undifferentiated myoblasts or as differentiated myotubes. Furthermore, immortalized cells generally remain very close in expression profile to their parent primary populations: the exception that we observed was linked to loss of normal profile in the primary cells rather than the immortalized cells. Thus, immortalization is protective of the characteristics of the primary population against the effects of senescence in cell culture.

A second concern was that we typically select clonal lines from within each immortalized population on the basis of their capacity to form morphologically well-defined myotubes, visualized by desmin immunostaining, thus selecting just one cell subtype from among the variation that was present in the original primary population. Our data would suggest that this does not lead to any systematic biases. Differentiated clones did not show any systematic over-expression of myogenic and muscle contractile genes relative to primary cells (Fig. 4).

## Conclusions

Immortalization using hTERT and cdk4 transgenes preserves and protects the natural biological variation of desmin-purified human primary myoblasts. Clones isolated from immortalized primaries are representative of their parent primary population and represent valuable research tools for the study of human neuromuscular disease.

## Methods

### Cell lines and media

Myogenic primaries and clonal lines of two healthy (CHQ and C25 and three DMD (DMD 6311, DMD 6594, and DMD 8036) subjects and CD56-negative cell lines (AB424, AB431, AB439, AB440, AB423, AB425, AB429, and AB451) of eight healthy subjects were cultured in proliferation medium (1 volume of M199, 4 volumes of Dulbecco’s modified Eagle’s medium (DMEM), 20% foetal bovine serum (Invitrogen), 50 μg/mL gentamicin (Invitrogen), 25 μg/mL fetuin, 0.5 ng/mL bFGF, 5 ng/mL EGF, 0.2 μg/mL dexamethasone, 5 μg/mL insulin) on matrigel-covered petri dishes (0.1 mg/mL, 1 mL per 19.5 cm^2^ dish area, 45 min, 37 °C), incubated at 37 °C, 5% CO_2_. Cells were differentiated in 1 volume of M199, 4 volumes of DMEM + 2% foetal bovine serum + 1 μL/mL gentamicin.

### Cell sorting/immunolabeling

Stocks of primary myogenic cells were purified by magnetic activated cell sorting using anti-CD56 (a specific marker of myoblasts) beads (MACS, Miltenyl Biotech). Purity before and after cell sorting was determined by immunolabelling (anti-desmin (1/100, clone D33, Dako) and anti-mouse IgG1 AlexaFluor 488 (1/400, LifeTechnologies™)). One hundred thousand cells were plated on a μ-dish 35-mm-high ibidi Treat (ibidi®) in proliferating medium. After 48 h (approx. 70% confluence), the cells were washed three times with PBS and were cultured in DMEM to induce myogenic differentiation. At day 5 of differentiation, cells were fixed and permeabilised with 200 μL 95% ethanol at 4 °C overnight, and blocked with 20% horse serum + *0.1% Triton-X 100* in PBS, 500 μL, 1 h, RT. Primary antibody anti-desmin (D33, IgG1, 1:100, Dako) and secondary antibody goat anti-mouse IgG1 AlexaFluor 488 (1:400, Invitrogen™) were used. Culture dishes were washed three times with PBS, counter-stained with 1 ug/ml DAPI for 1 min, RT, washed three times with PBS, and mounted with ibidi mounting medium (ibidi®). Five non-overlapping images were acquired with an Olympus IX70 and an Olympus UPlan FI 10×/0.30 Ph1 objective equipped with a Photomatics CoolSNAP™ HQ camera. Images were acquired using Metavue 7.5.6.0 software. The percentage of desmin-positive cells (desmin to DAPI cells) was calculated. Sorted cells were found to be of equivalent of higher purity in each case and subsequently used in cell cultures.

### RNA isolation

Cells were plated in four sets of 400000 in 78.5 cm^2^ petri dishes (Falcon) for each cell. Cells were switched to differentiation medium after 2 days of proliferation (approx. 70% confluence). After 9 days of differentiation, cells were scraped and collected in PBS *(*100 uL*)*. Cells were centrifuged at 13000 rpm for 10 min, and PBS supernatant removed. Each pellet was dissolved in TRIzol (1 mL). The RNA was subsequently isolated (PureLink RNA Mini Kit, using TRIzol® Reagent with the PureLink® RNA Mini Kit section of the standard protocol) with the RNA eluted in 30 μL H_2_O.

### RNA quality control

RNA quality and purity were determined using the Nanodrop 2000 (Thermo Scientific, nucleic acid setting) followed by quality control analysis (Agilent 2100 Bioanalyzer, Eukaryote Total RNA Nano assay, Agilent RNA 6000 Nano Kit, Agilent RNA Nano LabChip) to determine the RNA integrity number (RIN, 28S/18S ratio).

### Gene expression profiling

An aliquot of 150 ng of high-quality total RNA from each sample was used for mRNA expression profiling. Samples were analyzed using Illumina® Gene Expression BeadChip Array technology (Illumina, Inc., San Diego, CA). Reverse transcription for synthesis of the cDNA strand, followed by a single in vitro transcription (IVT) amplification, that incorporates biotin-labeled nucleotides, were performed with Illumina® TotalPrep™ -96 RNA Amplification Kit (Ambion, Austin, TX). 750 ng of the biotin-labeled IVT product (cRNA) was hybridized to HumanHT-12v4_BeadChip (Illumina, Inc., San Diego, CA) for 16 h, followed by washing, blocking, and streptavidin-Cy3 staining according to the Whole Genome Gene Expression Direct Hybridization protocol (Illumina, Inc., San Diego, CA). The arrays were scanned using HiScanSQ System and obtained decoded images were analyzed by GenomeStudio™ Gene Expression Module—an integrated platform for the data visualization and analysis (Illumina, Inc., San Diego, CA).

### Transcriptomic raw data treatment

Raw intensity values (Illumina idat files) of 94 samples were passed to the R lumidat library (https://github.com/drmjc/lumidat) to create an EListRaw class object compatible with further downstream analyses in R/Bioconductor. Two samples were excluded at this stage due to having zero proportion of expressed probes (all other samples had more than 40% of probes expressed). The limma library [39] was then used to carry out neqc normalization—this involves background correction followed by quantile normalization, using negative control probes for background correction and both negative and positive controls for normalization [40]. Probes were excluded if: (1) they had low bead numbers in at least one sample; (2) they were not expressed (expression *p* value < 0.01) in at least 16 arrays; (3) they were annotated as *no match* or *bad* quality according to the Bioconductor illuminaHumanv4.db library. This gave a total of 15342 probes for further analysis. The Bioconductor library arrayQualityMetrics was used for quality assessment of the arrays [41]. PCA was applied to the normalized values and visualized using R’s prcomp and plot functions, and the rgl library for 3D visualization. The R *pheatmap* library was used to produce heatmaps of selected genes. Linear regression for differential expression analysis was applied using limma [39]. Samples were grouped according to cell_type (grouping samples into myoblasts, myotubes, CD56-neg, and the C25 outlier), and this parameter was used to create a design matrix (~0 + cell_type), to which limma fitted a linear model for the normalized expression values. Limma’s *arrayWeights* function was used to weight each sample according to its estimated reliability by measuring how well the expression values for that array followed the linear model [42], limma’s *duplicateCorrelation function* was used to estimate the correlation between technical replicates (samples from the same subject, cell type, and clonal state, were considered as technical replicates) [43], and limma’s *eBayes* function was used to rank genes in order of evidence for differential expression, based on an empirical Bayes method [44].

The data are deposited in NCBI’s Gene Expression Omnibus and are accessible through GEO series accession number GSE79263.

### Functional analyses of transcriptomic data

Rank-based gene set enrichment tests were carried out using GSEA [45] on the 15342 quality-filtered, normalized, non-log_2_, gene expression values described above, applying default settings (e.g., permutations on phenotype, collapse genes to max of probesets) except that minimum overlap with gene sets was changed from 15 to 8 to allow for the small sizes of some muscle gene sets. Gene sets were taken from the muscle gene sets homepage (Muscle Gene Sets v2) http://sys-myo.rhcloud.com/muscle_gene_sets.php or from the Molecular Signatures Database (http://software.broadinstitute.org/gsea/msigdb) [45]. Gene set enrichment mapping was generated using Cytoscape [46] and Enrichment Map [47]. Enrichment tests were carried out on the most differentially expressed 800 genes by fold-change after filtering for FDR < 0.05, using Enrichr [48]. Heatmaps were created using the pheatmap R function (Raivo Kolde, 2015).

For the consensus set of genes that were differentially expressed in previously published studies of myoblast differentiation, we downloaded publicly available gene expression data from the Gene Expression Omnibus (GEO; http://www.ncbi.nlm.nih.gov/geo) database. Four studies were chosen that included murine and human myoblasts and myotubes at five or more days of differentiation: GEO series GSE10424, GSE11415, GSE24811, and GSE26145. For each study, signals were converted to expression levels using robust multi-array averaging [49] and custom chip definition files based on Entrez genes from the BrainArray resource [50] at http://brainarray.mbni.med.umich.edu (mgu74av2mmentrezgcdf 17.1.0 for GSE10424, mouse4302mmentrezgcdf 17.1.0 for GSE11415, mogene10stmmentrezgcdf 17.1.0 for GSE24811 and huex10stv2hsrefseqcdf 17.1.0 for GSE26145). Specifically, raw CEL files were downloaded and fluorescence signals were background adjusted, normalized using quantile normalisation and log_2_ expression values were calculated using median polish summarization. To identify differentially expressed genes for each study, the gene expression matrices were analysed with statistical analysis of microarray method (SAM) [51]. For each comparison, a two-class procedure was applied, and the percentage false discovery rate (FDR) was calculated. The FDR threshold was set to 0.05, and the 300 most upregulated genes in both groups in the comparison were selected for further analyses. All data analyses were performed using R program version 3.0.1, Bioconductor 2.12 libraries, and R statistical packages.

### Assay of Akt pathway activity and microtubule stability

Primary and immortalized myoblasts of lines C25 and DMD8036 were differentiated for 9 days as described above.

For Akt pathway activity, proteins were extracted in RIPA buffer containing phosphatase inhibitors. 15 μg of protein lysates were separated on 4–12% Bis-Tris gels, then transferred onto PVDF membrane (overnight, 4 °C, 15 V). After blocking the membrane for 1 h at RT with 5% milk diluted in TBS-Tween, membrane was incubated with primary antibodies from the Phospho-Akt Pathway Antibody Sampler Kit (Cell Signalling Technology) at 1:1000 dilution: Phospho-Akt (Ser473), Akt (pan), Phospho-c-Raf (Ser259), Phospho-GSK-3β (Ser9), Phospho-PTEN (Ser380), Phospho-PDK1 (Ser241), and Phospho-Akt (Thr308). Each of these is phospho-specific except Akt (pan) which targets all Akt protein independent of its phosphorylation state. Secondary antibody was anti-rabbit IgG (1:2000 dilution), HRP-linked. Bands were detected by SuperSignal™ West Pico Chemiluminescent Substrate (Thermo Fisher) and X-ray film. C25 and DMD8036 films received 10 and 2-minute exposures, respectively, and were scanned using a Xerox Colorqube 9303.

For microtubule stability, cells were cultured in duplicate dishes, and cDNA was synthesized from RNA using Transcriptor First Strand cDNA Synthesis Kit (Roche), and amplified by SYBR qPCR synthesis: denaturation 95 °C 8 min; amplification (50 cycles) 95 °C for 15 s, 52 °C for 25 s, 72 °C for 15 s; Tm (for melting curve) 95 °C for 5 s, 40 °C for 30 s then gradually up to 95 °C; cooling 40 °C for 30 s. Product was separated on 2% agarose gel with EtBr and detected by UV light.

## Additional file

Additional file 1: Supplemental data. (DOCX 1029 kb)

## Abbreviations

DAPI: 4′,6-diamidino-2-phenylindole
DMD: Duchenne muscular dystrophy
DMEM: Dulbecco’s modified Eagle’s medium
FDR: False discovery rate
FSHD: Facioscapulohumeral muscular dystrophy
GSEA: Gene set enrichment analysis
LGMD-2B: Limb girdle muscular dystrophy type 2B
PBS: Phosphate-buffered saline
PCA: Principal component analysis
