# Supplementary material for: Skeletal muscle characteristics are preserved in hTERT/cdk4 human myogenic cell lines
Source: Skelet Muscle. 2016 Dec 8;6:43. doi: 10.1186/s13395-016-0115-5 (PMC5146814; doi:10.1186/s13395-016-0115-5)
Supplement: Additional file 1: — Supplemental data. (DOCX 1029 kb) [file 13395_2016_115_MOESM1_ESM.docx]

**SUPPLEMENTAL DATA**

**
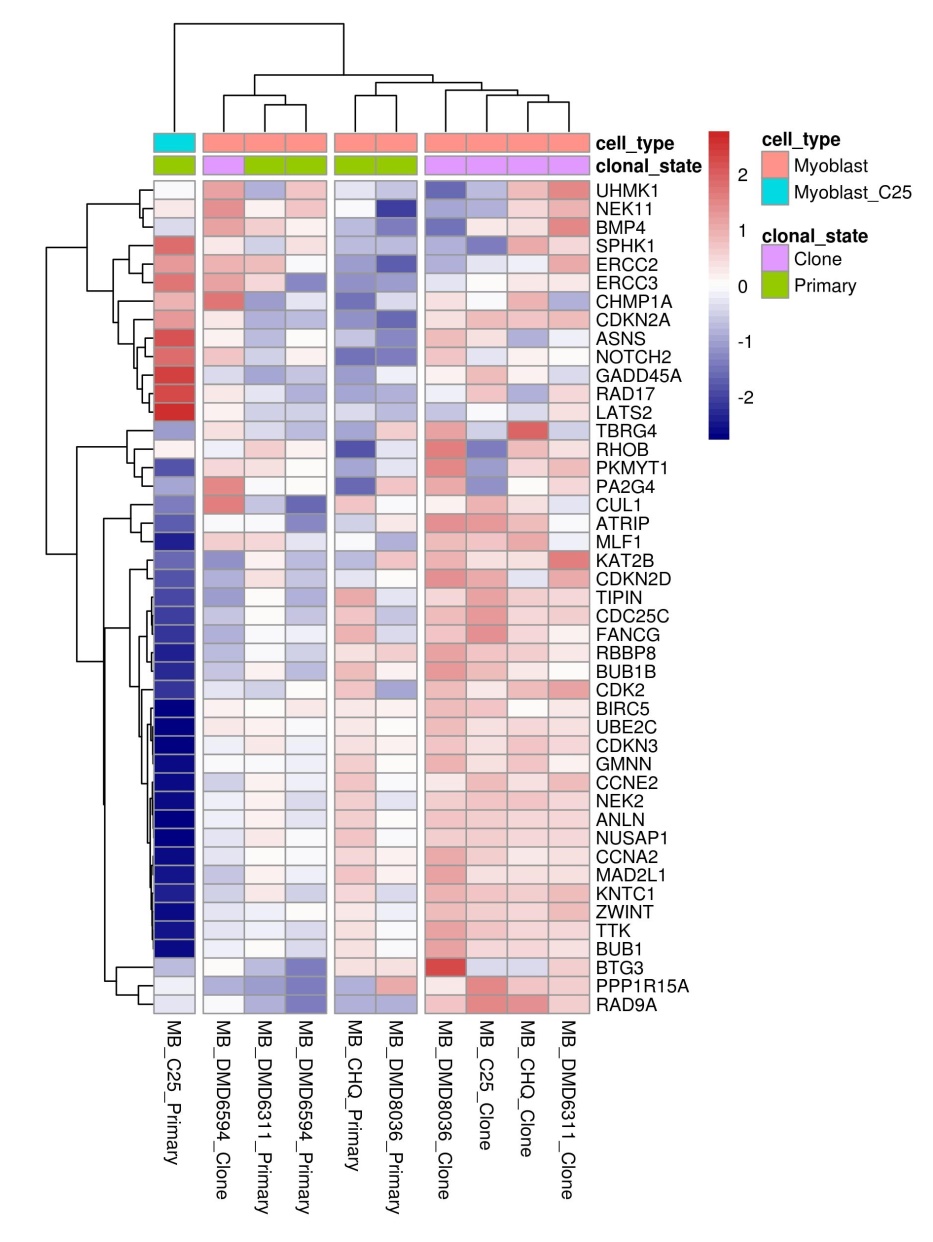
**

**Supplemental figure 1. Heatmap showing the expression levels in immortalized and primary human myoblasts of genes that may be mildly affected by immortalization. We specifically checked rank distribution patterns of 22 cell-cycle-related canonical pathways and gene ontologies. None of these were significantly enriched (all had FDR q-val > 0.2) but 6 had nominal p-values < 0.05 (a measure that does not adjust for gene set size or multiple hypothesis testing). As these can be considered borderline significant, we show here the expression levels of genes that were present in 3 or more of these 6 cell-cycle-related pathways/ontologies. These genes generally showed some upregulation in immortalized myoblast clones relative to primary myoblasts but with the C25 primary outlier showing relatively very strong dysregulation. Expression values are row scaled (i.e. changes are relative across each row for ease of interpretation). Hierarchical clustering analysis of the cell lines, indicated by branches at top, to some extent separates immortalized clones (green clonal_state) from their parent primary populations (green clonal_state). Hierarchical clustering analysis was also applied to genes (branches to left).**

**
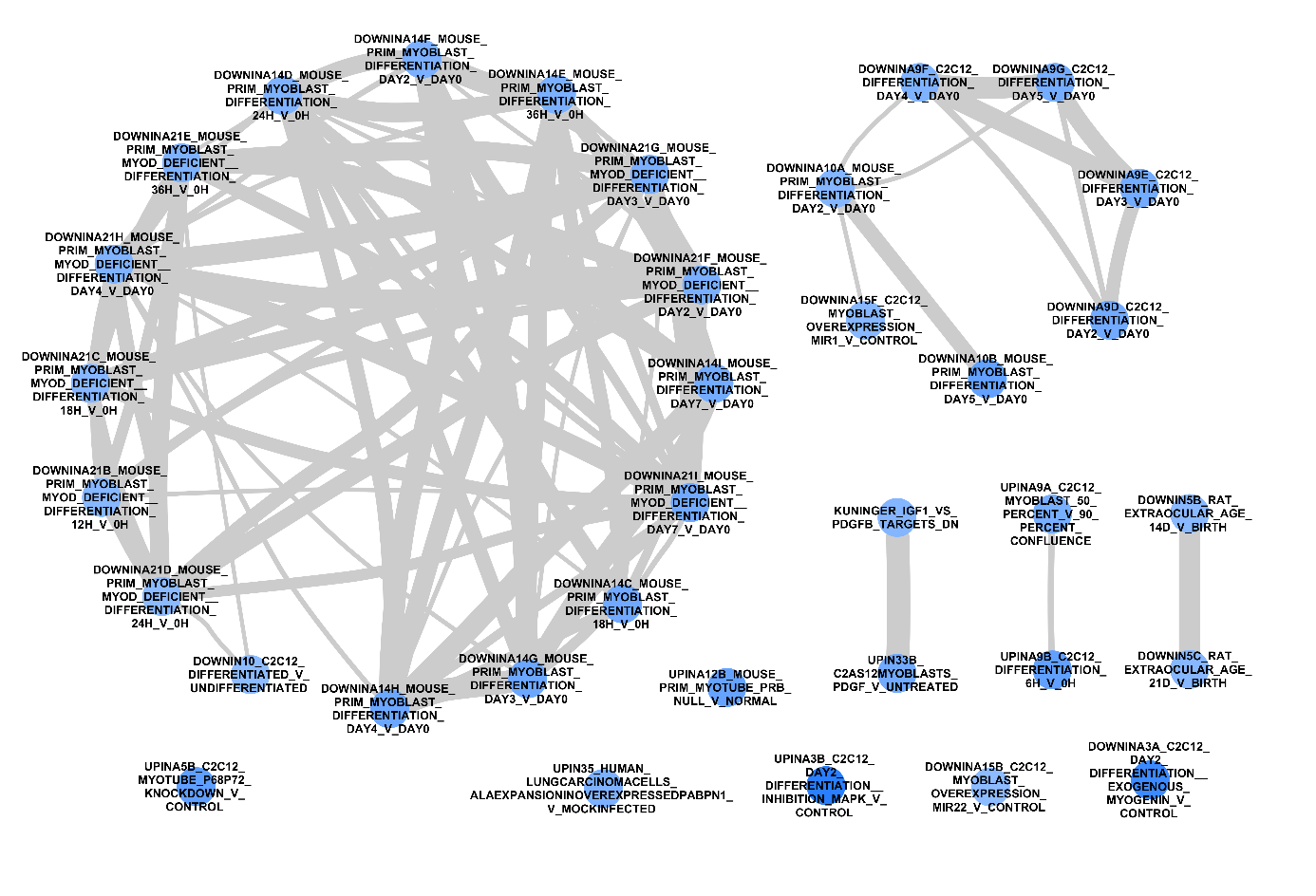
**

**Supplemental figure 2. Mapping of rank-based gene set enrichments. Each node represents a set of genes that was dys-regulated in a previously published muscle transcriptome study, and edges indicate sharing of the same genes between nodes (thicker edges indicates more overlap). The color of the node shows whether this gene set was up- or down-regulated (i.e. positively or negatively enriched – colored red or blue respectively – in this specific analysis, upregulated gene sets were not observed) in the comparison of myotubes v myoblasts in the present study. Included on this map are only gene sets having the most significant enrichment scores (FDR < 0.001) from among the 393 muscle gene sets that were tested. A similar comparison of immortalized v primary lines (tested independently for both myoblasts and myotubes) yielded no significant muscle gene sets, even at the weaker cut-off of FDR < 0.05.**

**
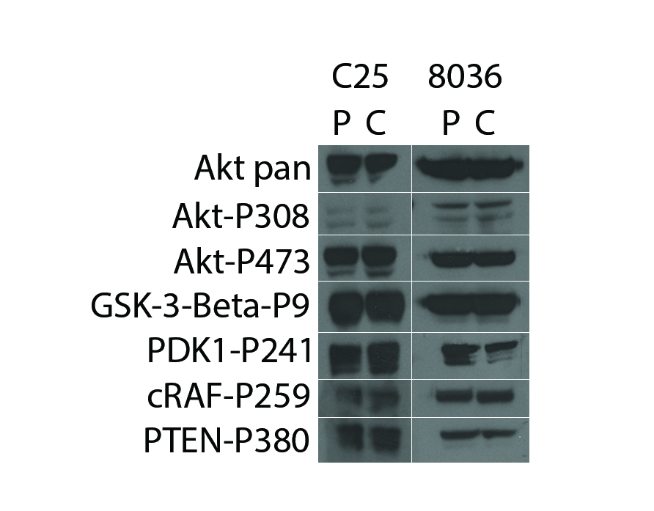
**

|  | **Intensity** | |
| --- | --- | --- |
|  | **C25** | **8036** |
| **Akt pan** | 0.943 | 0.993 |
| **Akt-P308** | 1.007 | 1.016 |
| **Akt-P473** | 1.017 | 0.984 |
| **GSK-3-Beta-P9** | 1.008 | 0.984 |
| **PDK1-P241** | 1.005 | 0.916 |
| **cRAF-p259** | 1.020 | 1.001 |
| **PTEN-P380** | 1.003 | 0.961 |

**Supplemental figure 3. Proteins involved in the regulation of Akt pathway activity. Lysates from differentiated cells of C25 and DMD8036 primary (labelled P) and immortalised (labelled C) cell lines. The antibodies used were those provided by the Phospho-Akt Pathway Antibody Sampler Kit (Cell Signalling Technology). They target Akt protein levels (Akt pan), and its activation at two phosphorylation sites (Akt-P308 and Akt-P473), as well as PTEN (a major negative regulator if the Akt pathway), GSK-3beta (regulated by Akt and involved in glycogen synthesis), c-Raf (regulated by Akt and involved in apoptosis), and PDK1 (an activator of Akt). Ratios of immortalized to primary band intensities are given (lower panel). The primaries and clones of the individual cell lines displayed similar levels of the detected proteins.**

**A**
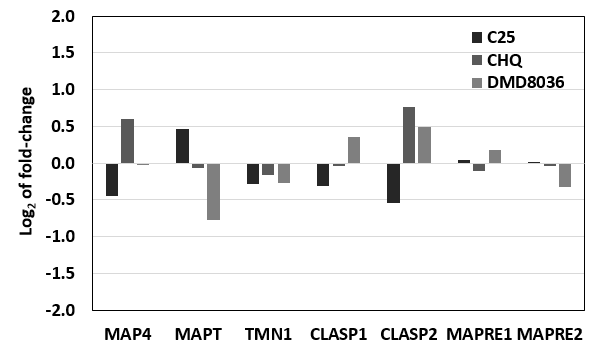


**B
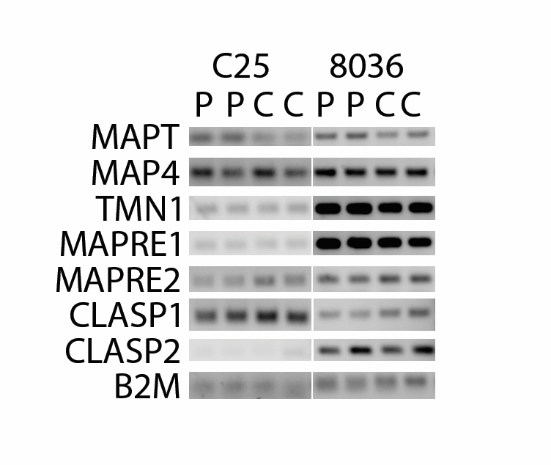
**

**Supplemental figure 4. Expression levels of genes involved in microtubule stability. (A) Log_2_ of fold-change (i.e. a value of 1 is equivalent to a 2-fold change in gene expression) for immortalized v primary myotubes (average of duplicate cultures) of lines C25, CHQ, and DMD8036. (B) Images of agarose gels showing PCR product amplified from cDNA synthesized from RNA isolated from differentiated cells of C25 and DMD8036 primary (labelled P) and immortalized (labelled C) cell lines. These genes encode microtubule interacting proteins (CLASP1, CLASP2, MAP4, MAPT, and STMN1); and/or are involved in microtubule organization and biogenesis (CLASP1, MAPT, and STMN1), in polymerization/depolymerisation (CLASP1, CLASP2, MAP4, MAPRE1, MAPRE2, MAPT, and STMN1), or in spindle organization and biogenesis (CLASP1, CLASP2, MAPRE1). The myotubes were cultured in duplicate dishes, and band intensities show no differences between primary and immortalized populations.**


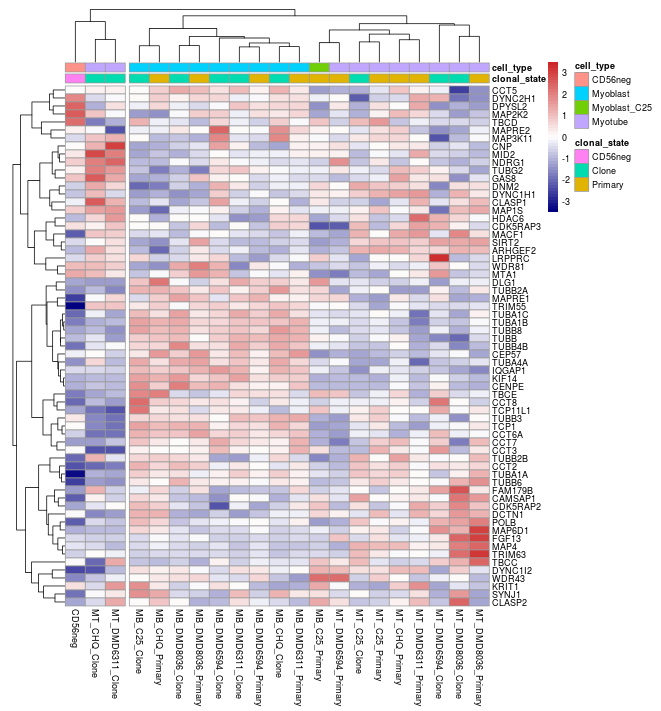


**Supplemental figure 5. Heatmap of gene expression for genes manually annotated to the microtubule term of the Gene Ontology, including genes assayed by PCR above. Hierarchical clustering analysis of the cell line is indicated by branches at top, and was also applied to genes (branches to left).**


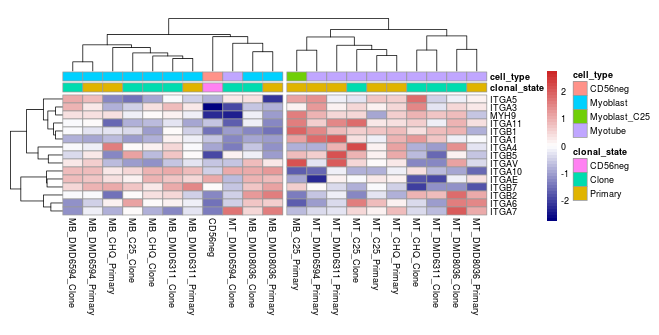


**Supplemental figure 6. Heatmap of gene expression for genes annotated to the integrin complex term of the Gene Ontology. Hierarchical clustering analysis of the cell line is indicated by branches at top, and was also applied to genes (branches to left).**


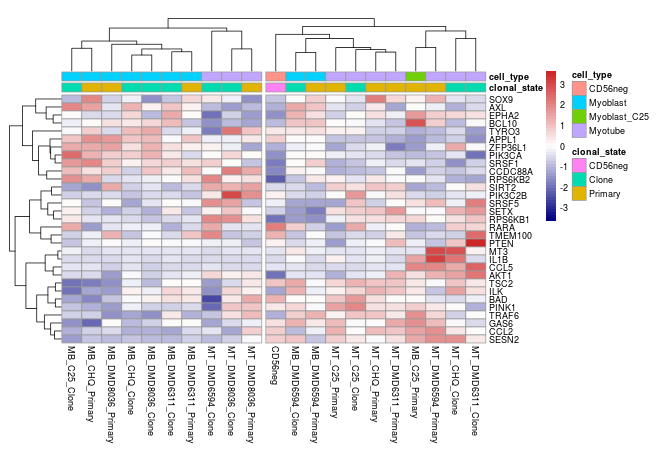


**Supplemental figure 7. Heatmap of gene expression for genes annotated to the protein kinase B binding (i.e. Akt binding) term of the Gene Ontology. Hierarchical clustering analysis of the cell line is indicated by branches at top, and was also applied to genes (branches to left).**

**C25 v myoblast**


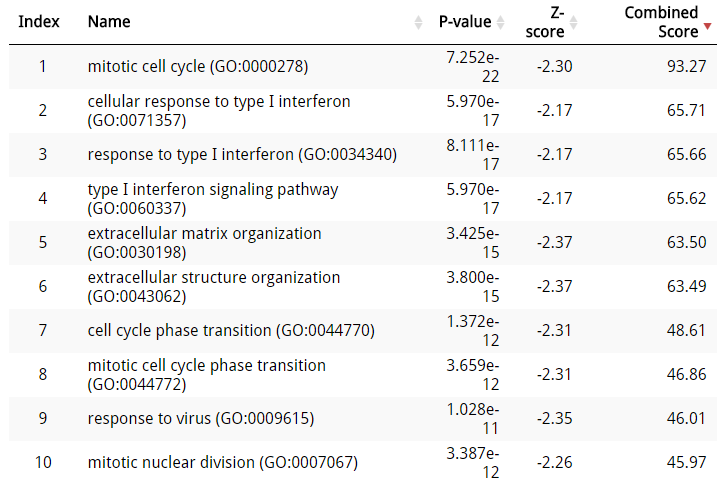


**C25 v myotube**


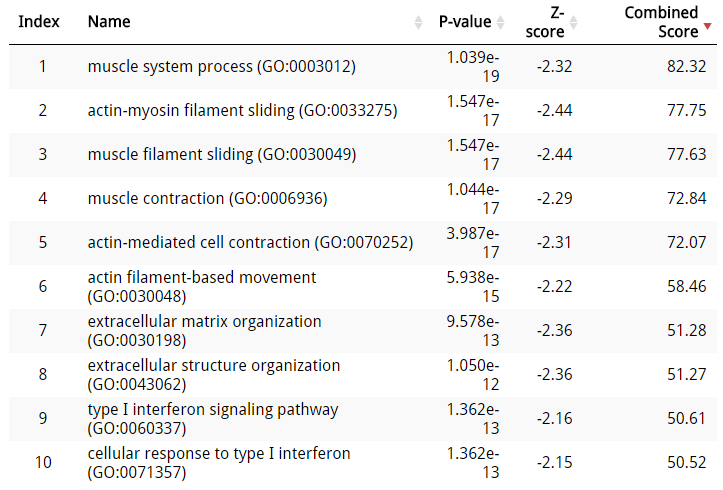


**Supplemental figure 8. Over-representation analysis of differentially expressed genes shows that the primary change in C25 primary myoblasts v other myoblasts is in the down-regulation of cell cycle processes (upper table), and in C25 primary myoblasts v myotubes it is the down-regulation of muscle contractile components (lower table). Enrichment of gene ontology biological processes among the top 800 by fold-change of genes having FDR < 0.05.**

| **GEO series** | **Platform** | **GEO samples** | **Reference** |
| --- | --- | --- | --- |
| GSE11415 | Mouse Genome 430 2.0 | \| GSM288014 \| \| --- \| \| GSM288015 \| \| GSM288016 \| \| GSM288032 \| \| GSM288033 \| \| GSM288034 \| | 1 |
| GSE24811 | Mouse Gene 1.0 ST | \| GSM610848 \| \| --- \| \| GSM610849 \| \| GSM610850 \| \| GSM610854 \| \| GSM610855 \| \| GSM610856 \| | 2 |
| GSE26145 | Human Exon 1.0 ST | \| GSM443913 \| \| --- \| \| GSM443914 \| \| GSM443915 \| \| GSM443916 \| \| GSM443917 \| \| GSM443918 \| | 3 |
| GSE10424 | Murine Genome U74A Version 2 | \| GSM263671 \| \| --- \| \| GSM263672 \| \| GSM263673 \| \| GSM263674 \| \| GSM263675 \| \| GSM263676 \| \| GSM263677 \| \| GSM263678 \| \| GSM263679 \| \| GSM263752 \| \| GSM263753 \| \| GSM263754 \| \| GSM263755 \| \| GSM263756 \| \| GSM263757 \| \| GSM263758 \| \| GSM263759 \| \| GSM263760 \| | -- |
|  |  |  |  |

**Supplementary Table 1. Complete list of gene expression datasets used in this study and their sources.**

**References to Supplementary Table 1**

1. Ma Q, Chirn GW, Szustakowski JD, Bakhtiarova A, Kosinski PA, Kemp D, Nirmala N. Uncovering mechanisms of transcriptional regulations by systematic mining of cis regulatory elements with gene expression profiles. BioData Min. 2008 Jul 17;1(1):4.
2. Soleimani VD, Yin H, Jahani-Asl A, Ming H, Kockx CE, van Ijcken WF, Grosveld F, Rudnicki MA. Snail regulates MyoD binding-site occupancy to direct enhancer switching and differentiation-specific transcription in myogenesis. Mol Cell. 2012 Aug 10;47(3):457-68.
3. Tsumagari K, Chang SC, Lacey M, Baribault C, Chittur SV, Sowden J, Tawil R, Crawford GE, Ehrlich M. Gene expression during normal and FSHD myogenesis. BMC Med Genomics. 2011 Sep 27;4:67.
